# SLEEPLESS SUPPORT: CAREGIVER SLEEP QUALITY AND ITS CORRELATES

**DOI:** 10.1093/geroni/igae098.3526

**Published:** 2024-12-31

**Authors:** Alexander Erickson, Susan McCurry, Monica Kelly, Anna Papazyan, Karen Camacho, Michael Mitchell, Jennifer Martin, Yeonsu Song

**Affiliations:** VA Greater Los Angeles Healthcare System, Los Angeles, California, United States; University of Washington, Seattle, Washington, United States; Greater Los Angeles VA Health Care System, North Hills, California, United States; Greater Los Angeles VA Health Care System, North Hills, California, United States; Greater Los Angeles VA Health Care System, North Hills, California, United States; Greater Los Angeles VA Health Care System, North Hills, California, United States; VA Greater Los Angeles Healthcare System, Los Angeles, California, United States; University of California Los Angeles, Los Angeles, California, United States

## Abstract

Adult caregivers of people living with dementia (PLWD) experience poor sleep, negatively impacting their ability to take a caregiving role. This study explored associations between caregiver sleep quality and factors at caregiver and PLWD levels. Participants were 45 caregiver-PLWD dyads, with a majority identifying as female (n=36; 75%), non-Hispanic white (n=19;39%), and having some college education (n=39; 87%). Caregiver sleep quality was measured with the Pittsburgh Sleep Quality Index (PSQI global score and three subscales: Sleep Efficiency, Perceived Sleep Quality, and Daily Disturbances). Other measures included the Zarit Burden Interview (ZBI), the Revised Memory and Behavior Problems Checklist (RMBPC) frequency and caregiver distress subscales, PLWD activities of daily living (ADLs), and PLWD Sleep Disorders Inventory (SDI) total score. Analyses included descriptive statistics, Pearson correlations, and t-tests. On average, caregivers endorsed poor global sleep quality (PSQI; mean of overall score). Higher caregiver Daily Disturbances scores were associated with higher ZBI scores (r=.33, p=.03), lower PLWD’s ADL functioning (r=-.35, p<.001), higher RMBPC frequency (r=.31, p=.04), and higher SDI (r=.39, p=.008). Higher caregiver age was associated with better PSQI global sleep quality (r=-.35, p=.02) and lower Daily Disturbance subscale scores (r=-.49, p<.001). Poor Sleep Efficiency subscale scores were associated with more sleep problems of PLWD (r=.38, p=.01), and caregivers of non-White race had significantly worse sleep efficiency (t(43)=3.17, p=.003). Caregivers of PLWD generally reported poor sleep, which was associated with caregiver burden, care recipient function and behavioral impairments. Addressing PLWD’s sleep and dementia-related behavioral challenges may help caregivers improve their sleep quality.

